# Supplementary material for: CARD8 inflammasome activation triggers pyroptosis in human T cells
Source: EMBO J. 2020 Aug 25;39(19):e105071. doi: 10.15252/embj.2020105071 (PMC7527815; doi:10.15252/embj.2020105071)
Supplement: Supplementary file 1 — Expanded View Figures PDF [file EMBJ-39-e105071-s001.pdf]

Expanded View Figures

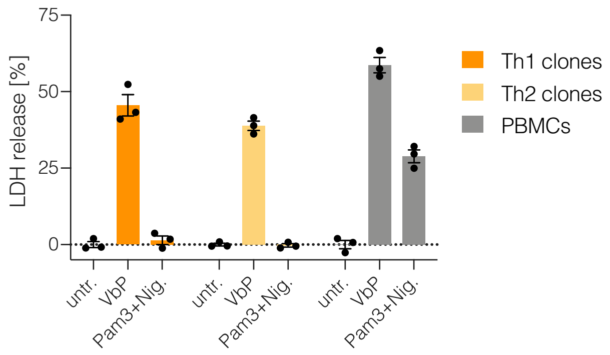

Figure EV1. CD4 T cell clones retain susceptibility toward VbP.

Naïve CD4 T cells were activated with CD3/CD28 beads in the presence of IL-2. On day 2, cells were subjected to minimal dilution cloning. When colonies became visible, clones were collected, cell numbers assessed and cells re-plated for stimulation. Forty thousand cells of each clone were stimulated with PMA/Ionomycin for 24 h and concentration of IFNγ and IL-4 was determined by ELISA. Based on the cytokine-profile, clones were identified as Th1 or Th2. Th1 and Th2 clones from one donor were then pooled and subjected to the indicated treatments. Freshly isolated PBMCs from an unrelated donor were stimulated in parallel. Cytotoxicity was determined by LDH assay. Individual data points ± SEM of three biological replicates from one donor.

© 2020 The Authors

© 2020 The Authors

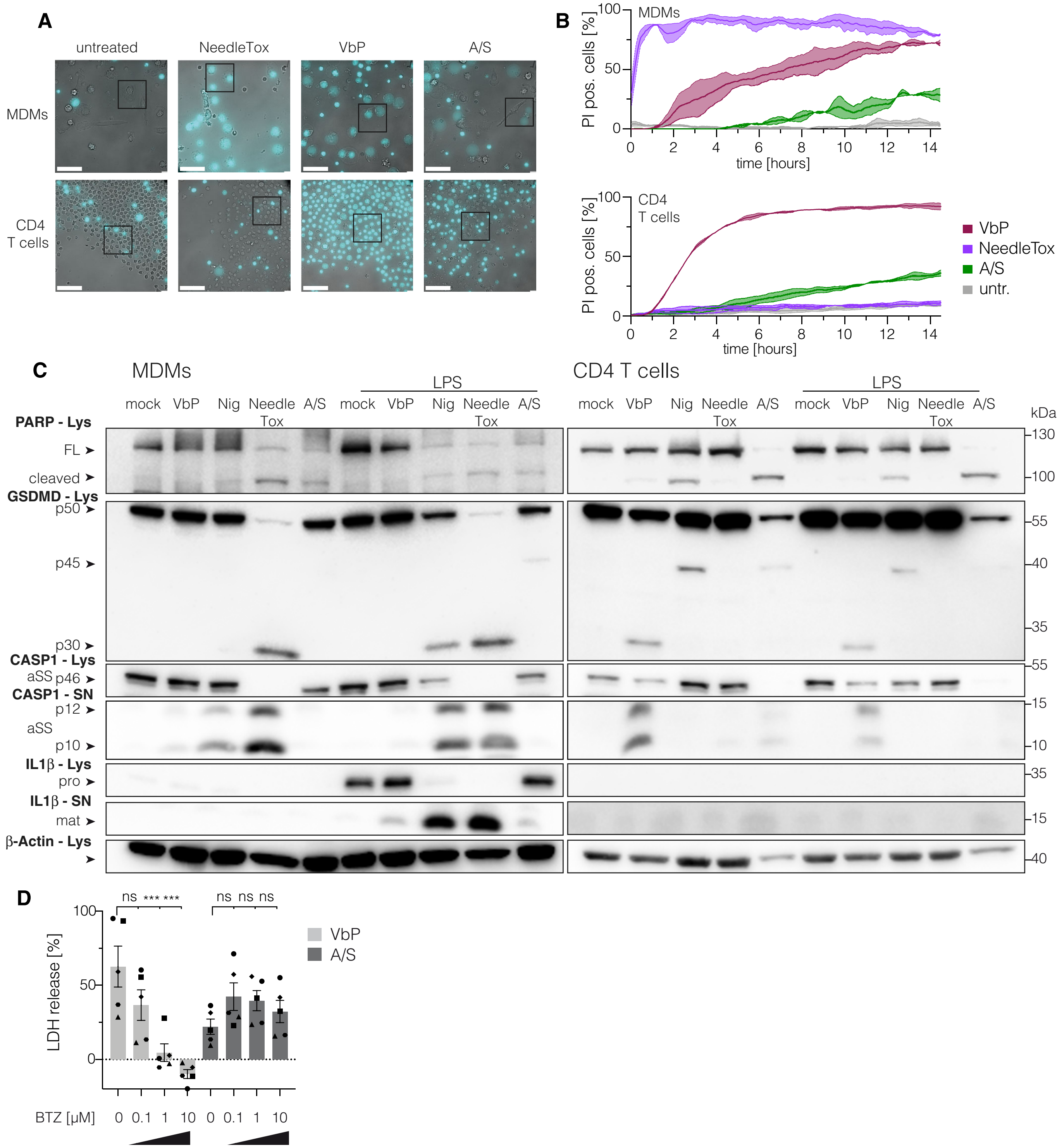

Figure EV2.

© 2020 The Authors

Figure EV2. Proteasome-inhibition blocks VbP-induced pyroptosis in CD4 T cells.

A Uncropped and less magnified versions of the images displayed in Fig 2B. Scale bars: 100 μm.

B Quantification of PI-positive cells of data shown in Movies EV1–EV6 and Fig 2A and of an additional donor. Data were normalized by a sliding window covering 30 min. Data (mean ± SEM) from two independent donors are shown.

C CD4 T cells or MDMs from one donor were subjected to the indicated treatments: Nigericin (Nig. 4 h), NeedleTox (4 h), Val-boroPro (VbP 22 h), and ABT737/S63845 (A/S 22 h). When indicated, cells were primed with LPS for 2 h prior to stimulation. αSS and αLS indicate the use of a small subunit- and a large subunit-specific CASP1 antibody, respectively. Lys = lysate, SN = supernatant, FL = full-length protein, pro = proIL-1β, mat = mature IL-1β. Shown is one representative experiment out of three independent experiments.

D CD4 T cells were treated as indicated for 8 h and cytotoxicity was determined by LDH assay. Individual data points ± SEM from five independent donors are shown. Statistics indicate significance by two-way ANOVA: $^{***}P \leq 0.001$; ns, not significant. *P*-values were corrected for multiple comparisons (Dunnett).

© 2020 The Authors

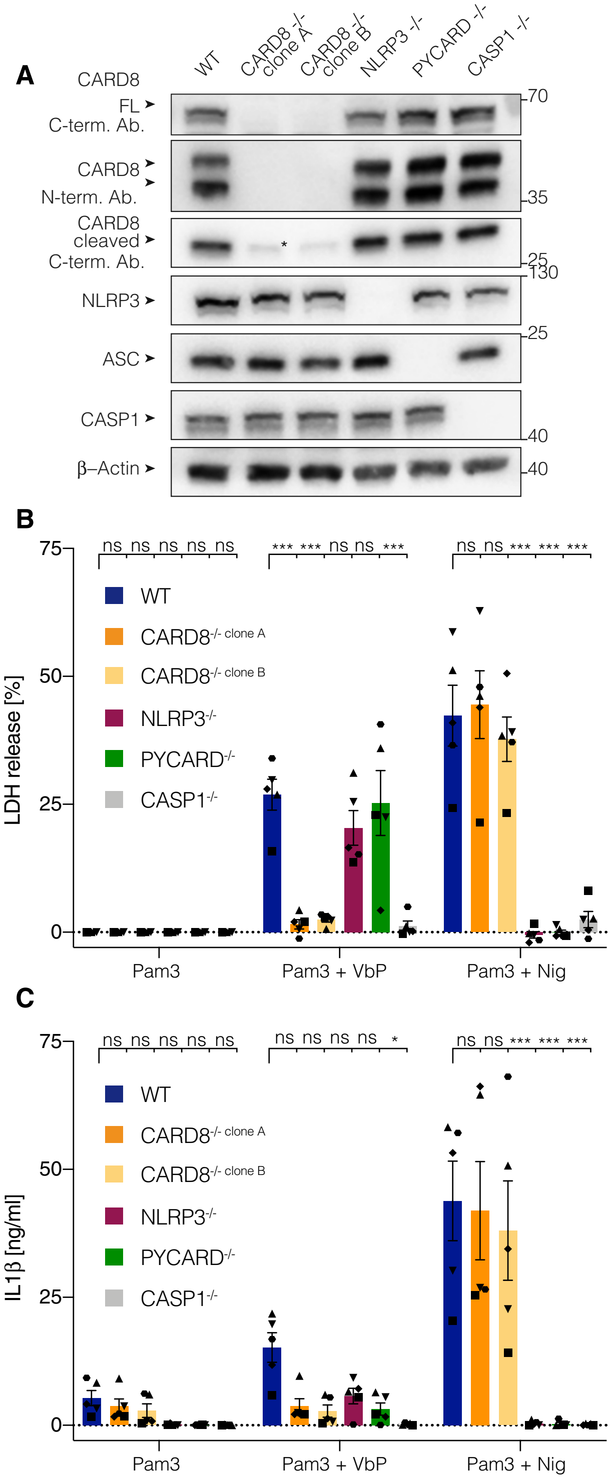

Figure EV3. VbP triggers CARD8-dependent pyroptosis in human myeloid cells.

A Immunoblotting of clonal THP-1 knockout cell lines of the indicated genotype. * indicates a band at the size of the cleaved CARD8 c-terminal fragment that was still detectable in the CARD8 knockout cell lines, either indicating unspecific banding or the existence of an isoform that was not affected by the gene targeting approach. One representative blot of three is shown.

B, C THP-1 cells of the indicated genotype treated with either Pam3CSK4 alone (Pam3, 24 h), Pam3CSK4 (1 h priming) and Val-boroPro (VbP 23 h), or Pam3CSK4 (4 h priming) and Nigericin (Nig., 4 h). LDH-activity (B) and IL-1β concentration (C) in the supernatant were determined by LDH cytotoxicity assay and ELISA, respectively. Individual data points ± SEM from five independent experiments are shown. Statistics indicate significance by two-way ANOVA: ***$P \leq 0.001$; *$P \leq 0.05$; ns, not significant. *P*-values were corrected for multiple comparisons (Dunnett).

© 2020 The Authors

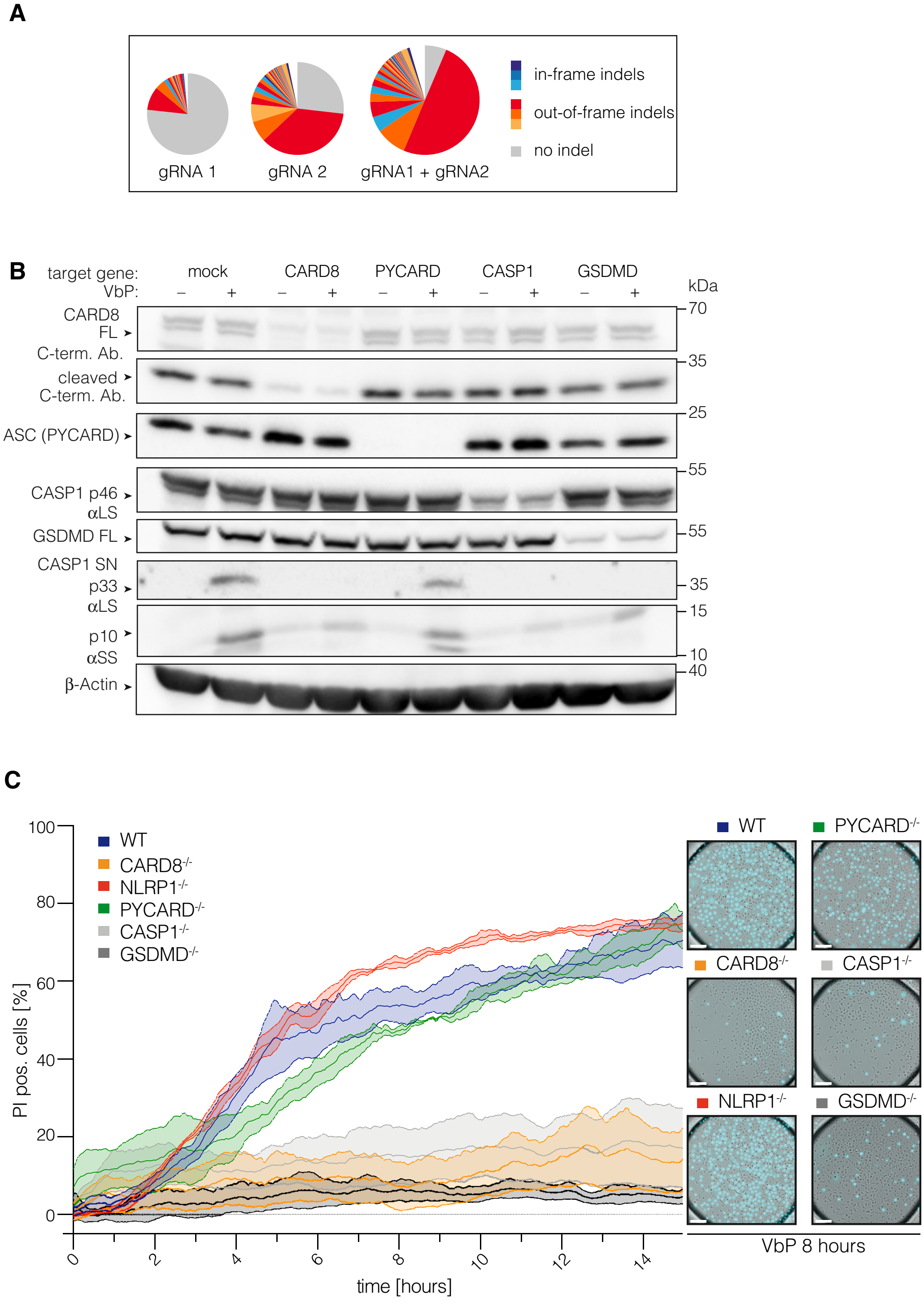
A
gRNA 1
gRNA 2
gRNA1 + gRNA2
in-frame indels
out-of-frame indels
no indel
B
target gene:
VbP:
mock
CARD8
PYCARD
CASP1
GSDMD
kDa
CARD8
FL
C-term. Ab.
cleaved
C-term. Ab.
ASC (PYCARD)
CASP1 p46
αLS
GSDMD FL
CASP1 SN
p33
αLS
p10
αSS
β-Actin
70
35
25
55
55
35
15
10
40
C
WT
CARD8-/-
NLRP1-/-
PYCARD-/-
CASP1-/-
GSDMD-/-
PI pos. cells [%]
time [hours]
VbP 8 hours

Figure EV4.

Figure EV4. Polyclonal and clonal gene targeting approaches uncover a functional CARD8 inflammasome in T cells.

A MiSeq analysis of polyclonal CD4 T cells targeted either with CARD8 gRNA1, CARD8 gRNA2, or the combination of both.

B Immunoblot of VbP-treated CD4 T cells targeted with gRNAs against the indicated genes. Two gRNAs/target gene were used. 14 days after editing and expansion, cells were treated for 18 h with VbP.

C Clonal knockout T-cell pools of the indicated genotype were treated with VbP and morphologic changes as well as PI uptake were monitored by live-cell imaging microscopy using a 25× objective. Fraction of PI-pos. cells over time are shown. Images were acquired every 2 min. Background PI-levels from the start of the experiment were subtracted. Data were normalized by a sliding window covering 30 min. Data (mean ± SEM) from three independent experiments using the same clonal knockout T-cell pool from one donor are shown. On the right, representative images for each genotype at 8 h of VbP treatment are shown. Scale bars: 50 µm.

© 2020 The Authors
