# Supplementary material for: CARD8 inflammasome activation triggers pyroptosis in human T cells
Source: EMBO J. 2020 Aug 25;39(19):e105071. doi: 10.15252/embj.2020105071 (PMC7527815; doi:10.15252/embj.2020105071)
Supplement: Supplementary file 7 — Movie EV5 [file EMBJ-39-e105071-s007.zip › EMBOJ-2020-105071R1-Movie_EV5/MovieEV5_legend.docx]

**Expanded View Movie Legends**

**Movie EV5 – related to Figure 2A**

Time-lapse movies of live-cell imaging microscopy of cell types and treatments as indicated: (EV1) MDMs – NeedleTox, (EV2) CD4 T cells – NeedleTox, (EV3) MDMs – VbP, (EV4) CD4 T cells – VbP, (EV5) MDMs – ABT737/S63845, (EV6) CD4 T cells - ABT737/S63845, (EV7) MDMs – untreated, (EV8) CD4 T cells – untreated. Images were acquired every 2 minutes, movies are played at 25 frames/second. Cyan color coding is used for the fluorescent PI-signal. Scale bars: 25 µm.
