# Supplementary figures and images for: CARD8 inflammasome activation triggers pyroptosis in human T cells
Source: EMBO J. 2020 Aug 25;39(19):e105071. doi: 10.15252/embj.2020105071 (PMC7527815; doi:10.15252/embj.2020105071)

Figure EV3A

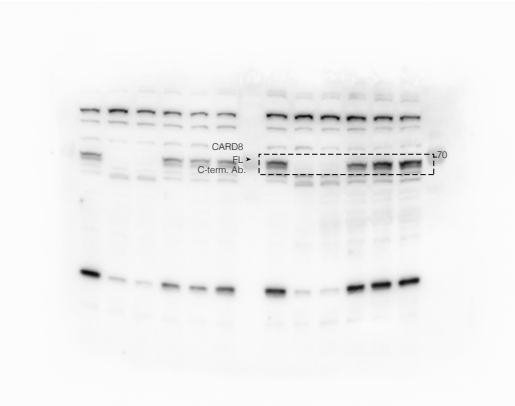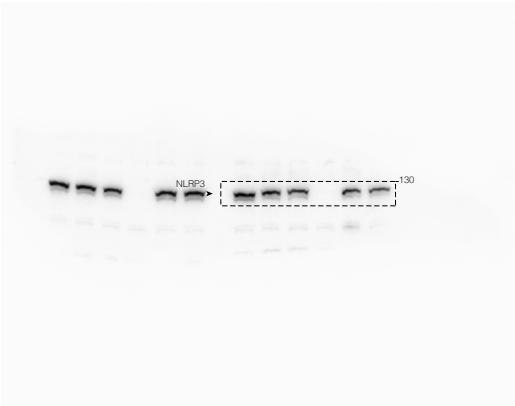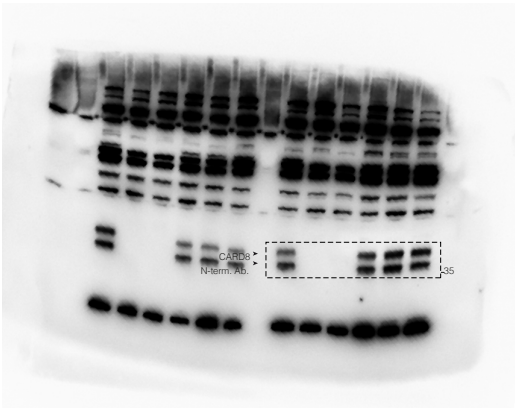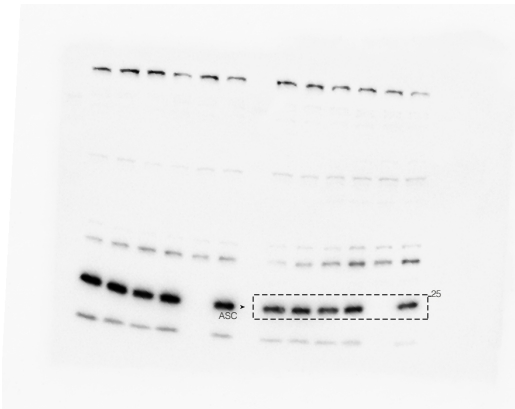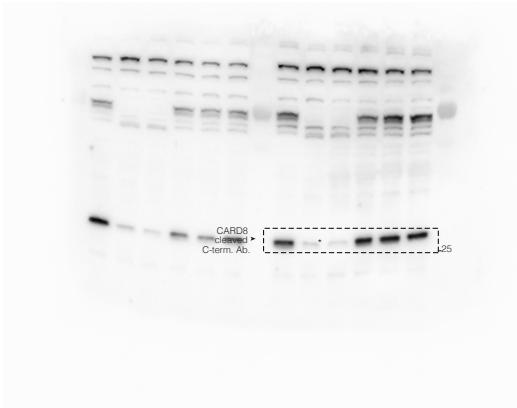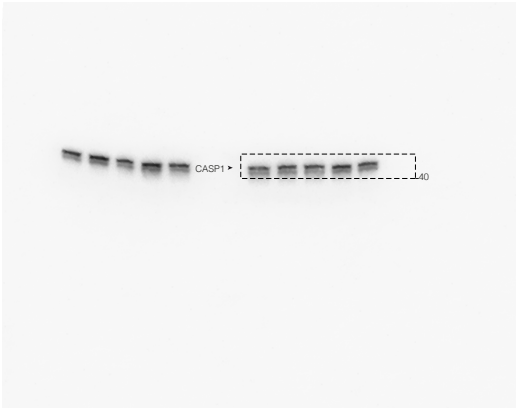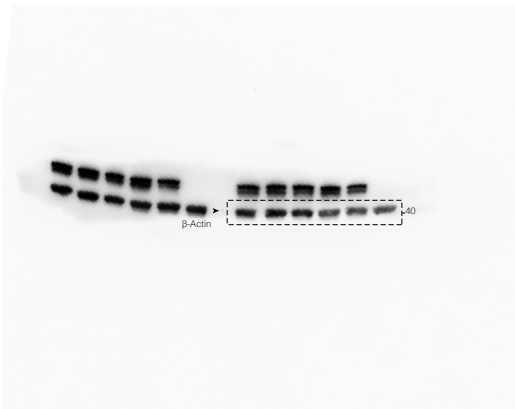

Supplement: Supplementary file 11 — Source Data for Expanded View [file EMBJ-39-e105071-s015.zip › EV_Figure_Source_Data/Figure_EV3_Source_Data-sd/Source data Figure EV3.pdf]

Figure EV4B

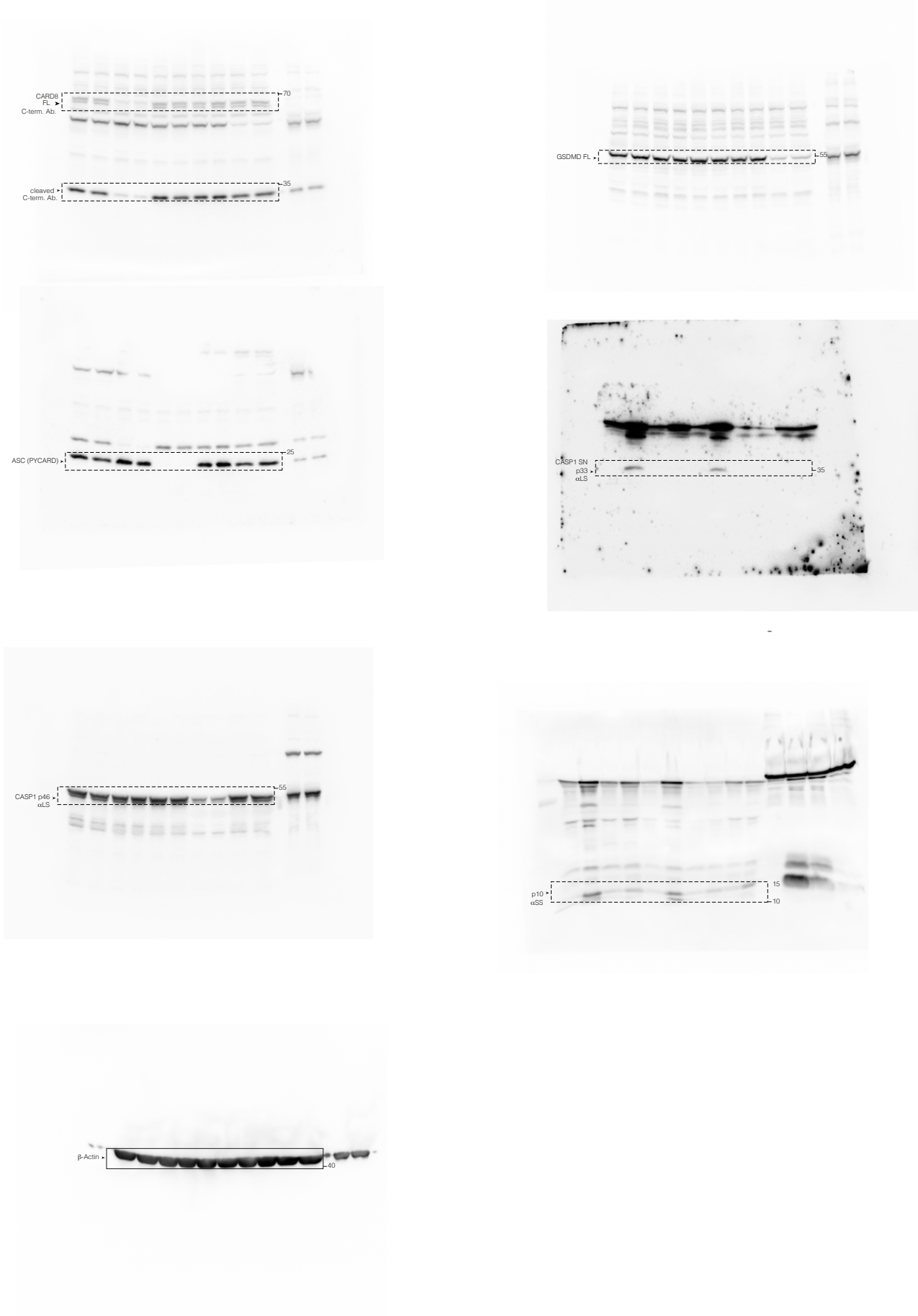

Supplement: Supplementary file 11 — Source Data for Expanded View [file EMBJ-39-e105071-s015.zip › EV_Figure_Source_Data/Figure_EV4_Source_Data-sd/Source data Figure EV4.pdf]

Figure EV2C

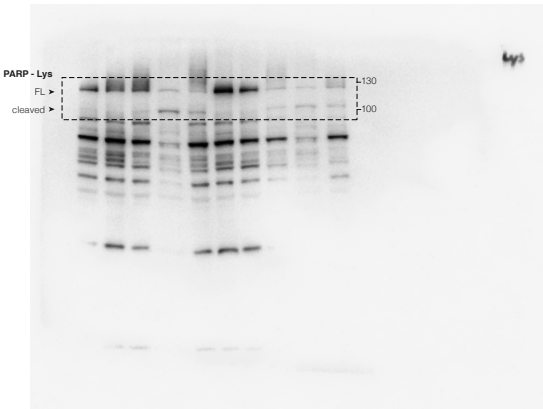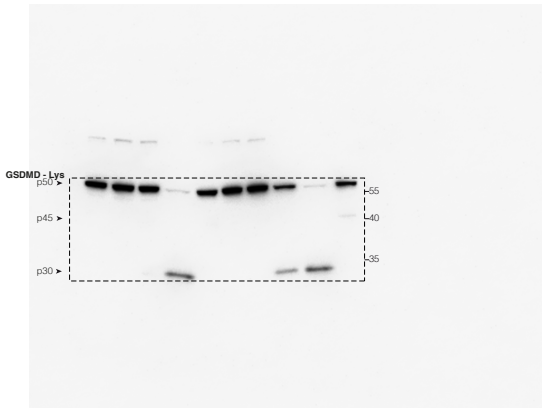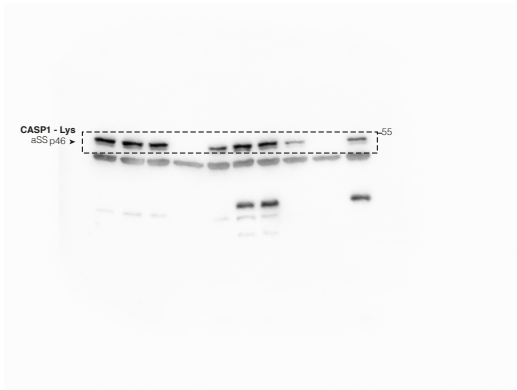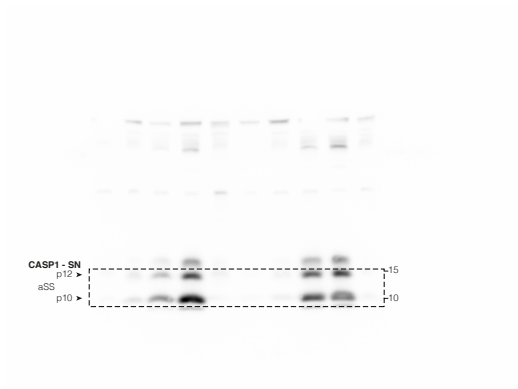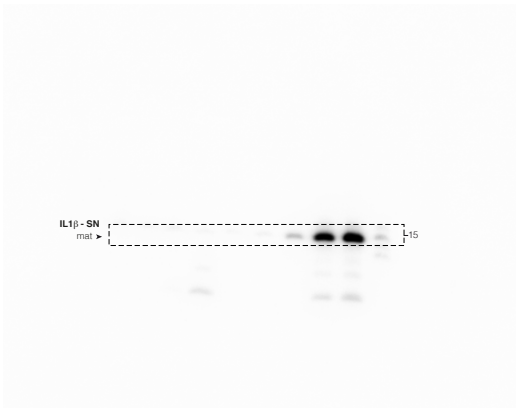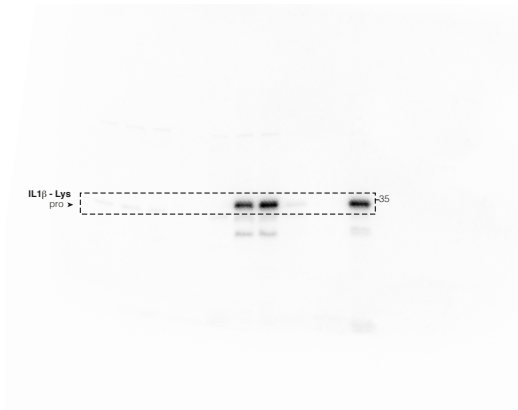

Figure EV2C

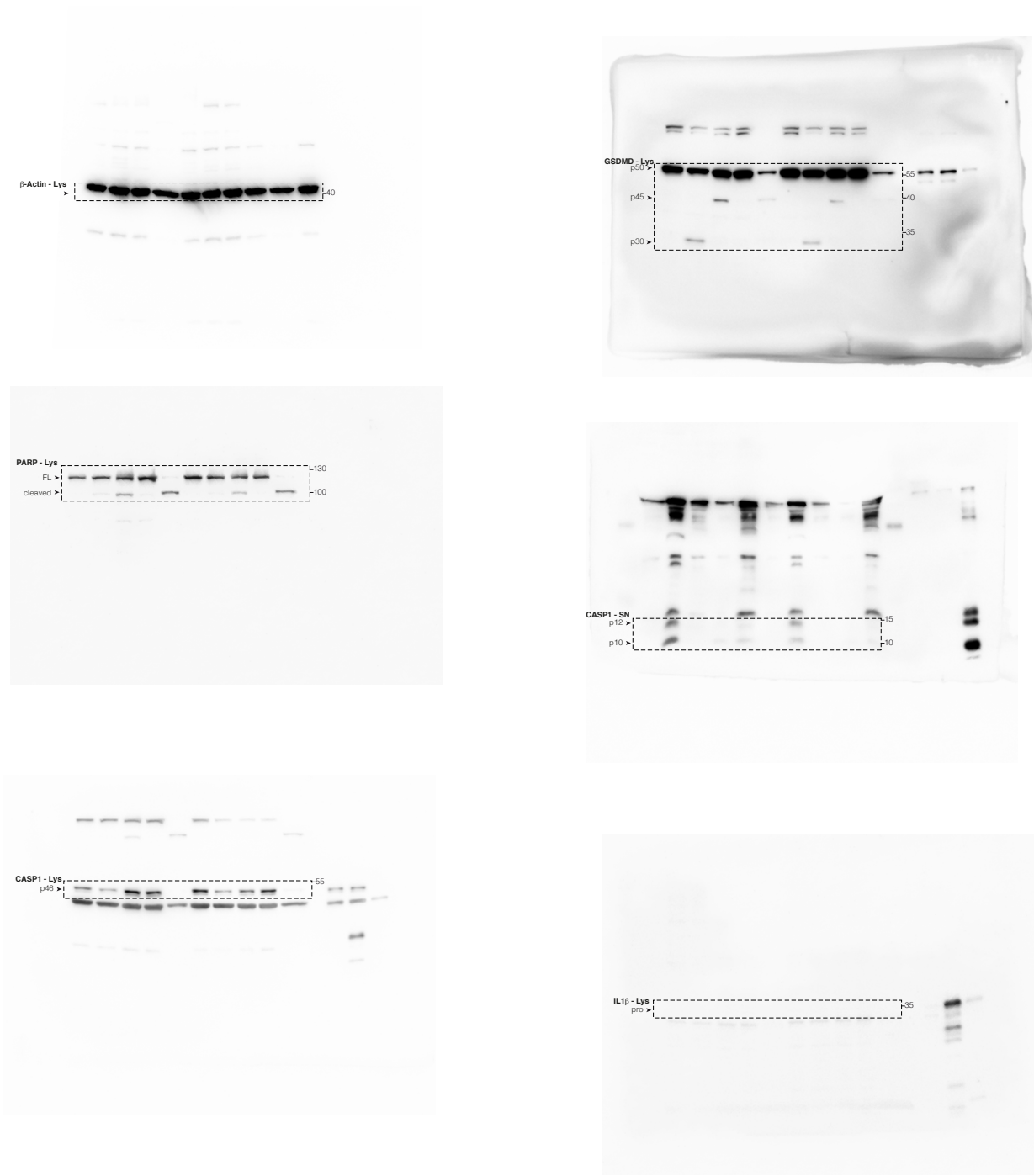

Figure EV2C

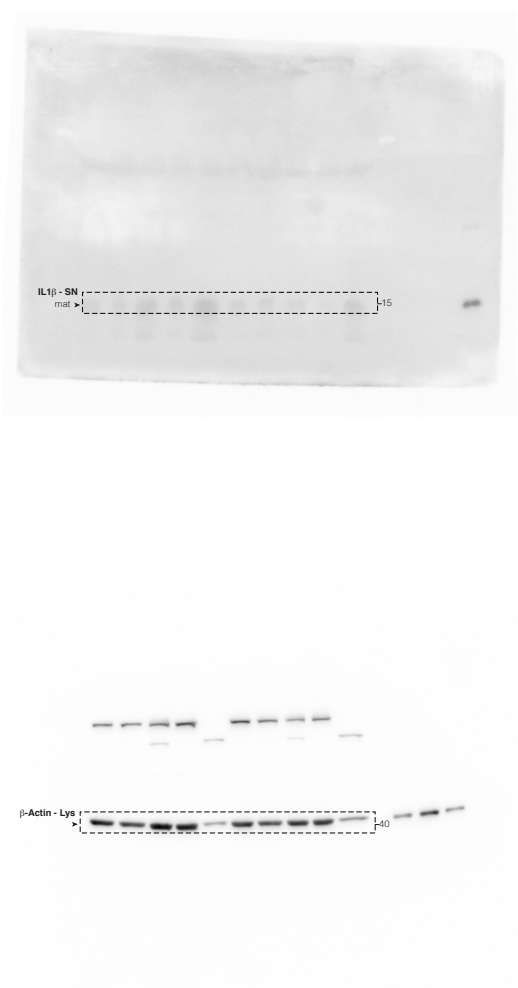

Supplement: Supplementary file 11 — Source Data for Expanded View [file EMBJ-39-e105071-s015.zip › EV_Figure_Source_Data/Figure_EV2_Source_Data-sd/Source data Figure EV2.pdf]

Figure 2C

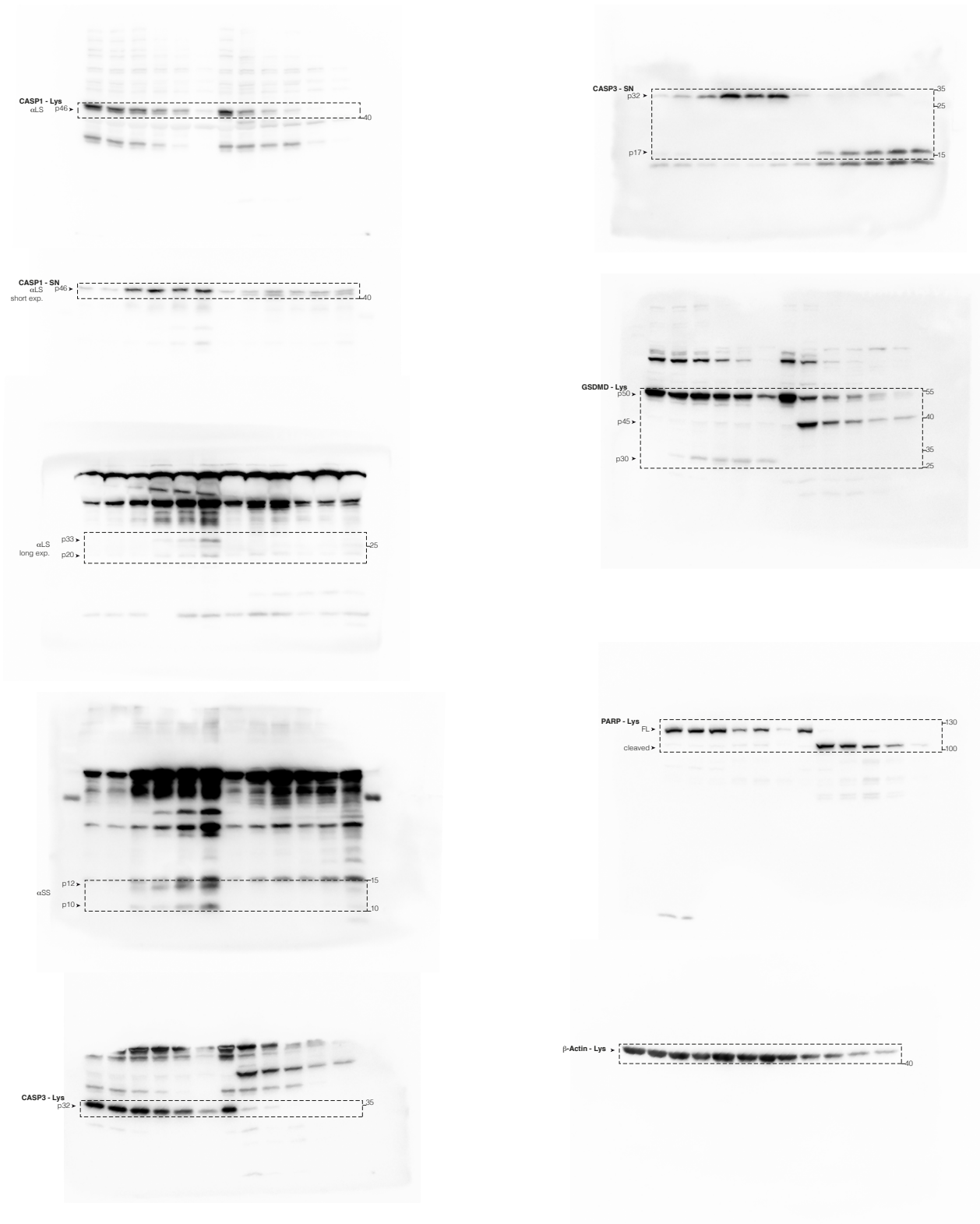

Supplement: Supplementary file 13 — Source Data for Figure 2 [file EMBJ-39-e105071-s011.pdf]

Figure 3B

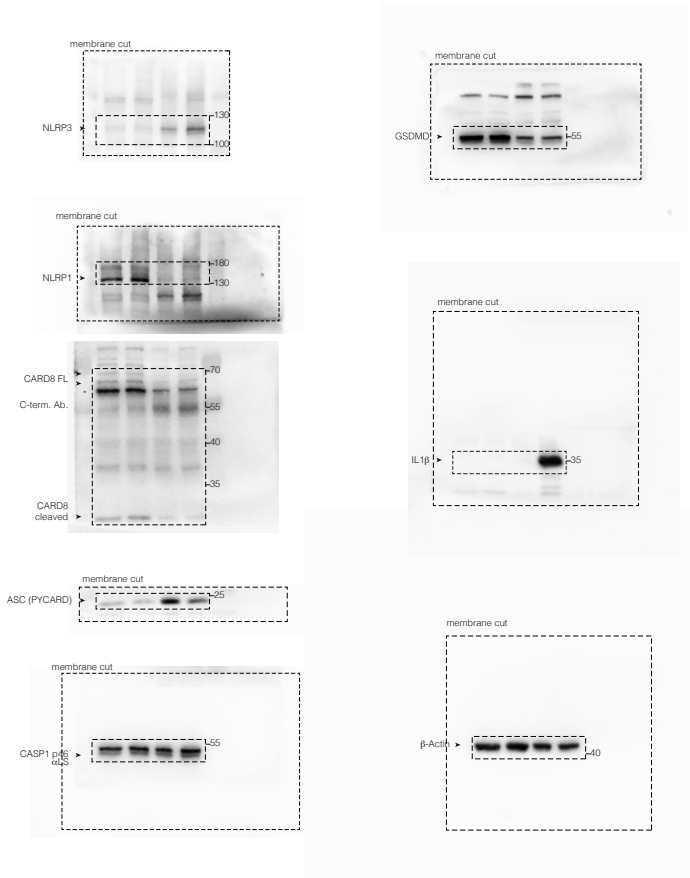

Figure 3C

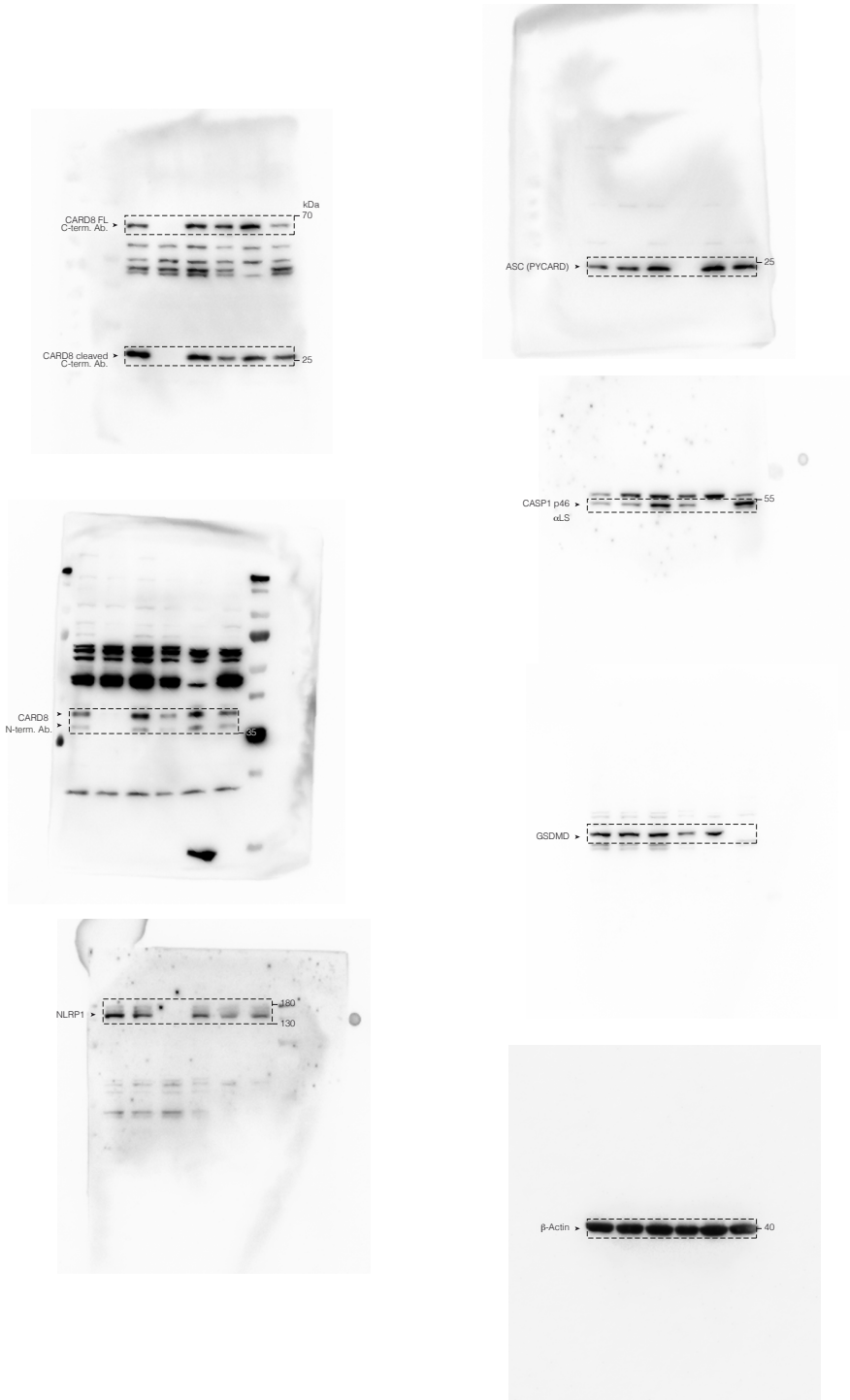

Supplement: Supplementary file 14 — Source Data for Figure 3 [file EMBJ-39-e105071-s012.zip › Source data Figure 3.pdf]

**Figure 5B**

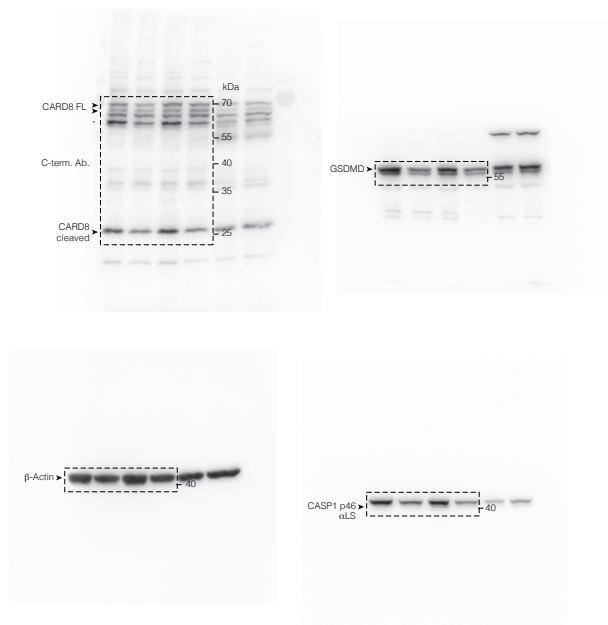

Supplement: Supplementary file 16 — Source Data for Figure 5 [file EMBJ-39-e105071-s014.zip › Source data Figure 5.pdf]
